# Comparison of the Greek Version of the Quick Mild Cognitive Impairment Screen and Montreal Cognitive Assessment in Older Adults

**DOI:** 10.3390/healthcare10050906

**Published:** 2022-05-13

**Authors:** Lambros Messinis, Grigorios Nasios, Antonios Mougias, Panayiotis Patrikelis, Sonia Malefaki, Vasileios Panagiotopoulos, Aikaterini Ntoskou Messini, Christos Bakirtzis, Nikolaos Grigoriadis, Panagiotis Ioannidis, Stella Bairami, Valentina Papadopoulou, Phillipos Gourzis

**Affiliations:** 1 School of Psychology, Laboratory of Cognitive Neuroscience, Aristotle University of Thessaloniki, 541 24 Thessaloniki, Greece; ppatrikelis@gmail.com; 2 Department of Psychiatry, University of Patras Medical School, 265 00 Patras, Greece; phgourzis@gmail.com; 3 Higher Educational Institute of Epirus, Department of Speech and Language Therapy, 453 32 Ioannina, Greece; grigoriosnasios@gmail.com; 4 Alzheimer Center of the Greek Psychogeriatric Association “Nestor”, 11144 Athens, Greece; amougias@gmail.com; 5 Department of Mechanical Engineering & Aeronautics, University of Patras, 265 04 Patras, Greece; sonia.malefaki@gmail.com; 6 Department of Neurosurgery, University Hospital of Patras, 265 00 Patras, Greece; panagiotopoulos2000@yahoo.com; 7 Second Department of Neurology, Multiple Sclerosis Center, Aristotle University of Thessaloniki, 541 24 Thessaloniki, Greece; katerinaalogotherapy@gmail.com (A.N.M.); bakirtzischristos@yahoo.gr (C.B.); ngrigoriadis@auth.gr (N.G.); ispanagi@auth.gr (P.I.); 8 School of Psychology, Postgraduate Program Clinical Neuropsychology, Aristotle University of Thessaloniki, 541 24 Thessaloniki, Greece; stellab97@gmail.com (S.B.); valentina8397@gmail.com (V.P.)

**Keywords:** cognitive screen, Qmci-Gr, MoCA, dementia, subjective cognitive decline, MCI

## Abstract

**Objective**: Cognitive screening instruments (CSIs) are essential for everyday practice. The Quick Mild Cognitive Impairment (Qmci) screen, a short instrument designed to identify mild cognitive impairment, was recently translated into Greek (Qmci-Gr). The present study compared its diagnostic value against the Montreal Cognitive Assessment (MoCA) screen and examined its optimal cutoffs. **Method**: We recruited consecutive patients aged ≥55 years that presented with cognitive complaints from two outpatient clinics in Greece. The Q*mci*-Gr and MoCA were completed by all patients. Furthermore, they were assessed independently with a comprehensive flexible neuropsychological battery to establish a diagnostic classification. **Results**: In the current study, we assessed a total of 145 patients, with a median age of 70 years; 44 were classified as having Subjective Memory Complaints (SMC) but normal cognition, 32 with MCI and 69 with dementia. The Qmci-Gr had a higher accuracy compared to the MoCA in discriminating MCI from dementia, area under the curve (AUC) of 0.81 versus 0.75, respectively; however, this finding was marginally significant (*p* = 0.08). Its accuracy was marginally higher for distinguishing SMC from dementia, AUC of 0.94 versus 0.89 (*p* = 0.03). However, Qmci-Gr presented a lower accuracy than MoCa in differentiating SMC from MCI, AUC of 0.76 versus 0.94 (*p* = 0.006). **Conclusions**: The Qmci-Gr has comparable diagnostic accuracy to the MoCA regarding MCI and dementia groups. Further research, with larger and more diverse samples, may be necessary to ensure generalizability.

## 1. Introduction

The ever-increasing number of people developing cognitive impairment and ultimately dementia [1], calls for the early identification of cognitive decline in older populations, ideally before the manifestation of objective cognitive impairment or brain changes, to prevent or delay the progression to dementia [2]. Longitudinal studies suggest that initiating and maintaining lifestyle changes and targeting dementia risk factors, contribute to either converting to normal cognition from the mild cognitive impairment status or maintaining this status without converting to dementia [3].

To improve treatment efficacy, early detection of cognitive impairment is crucial [4]. However, discriminating people presenting with subjective cognitive complaints from those with normal cognition is often challenging due to the lack of quantifiable cognitive decline [5]. Similarly, a differential diagnosis of Mild Cognitive Impairment (MCI) and dementia, although more attainable, remains difficult in clinical practice. Considering this, the improved accuracy of diagnostic tools is warranted to prevent a false diagnosis and to enable timely interventions [6].

Cognitive screening instruments (CSIs) offer practitioners the opportunity to briefly assess multiple cognitive domains in busy clinical environments [7]. Despite their practical use, CSIs have a number of limitations, including the impact of demographic and cultural determinants on total scores, the underassessment of several cognitive domains (e.g., specific executive dimensions) and a higher risk of false positive diagnosis, which can be prevented if further assessment is provided [8]. In addition, caution is called for, since most CSIs implemented in clinical practice present a low sensitivity and classification accuracy, while there is a considerable loss of valuable qualitative information when administered by less experienced staff. However, they provide information about a patient’s general cognitive ability in a short amount of time and have proven to be useful when combined with more comprehensive assessments [9]. The Mini Mental State Examination (MMSE) is widely use, but has a bias for higher education, e.g., cut off score of 29 for 70-year-olds with 12 years of education versus a cut-off score of 23 for 70-year-olds with 3 years of education. It also has a low ceiling effect (it is very easy) for highly educated persons and a high floor effect (it is very difficult) for demented patients in advanced stages. Furthermore, it does not pick up early cognitive losses or MCI. On the other hand, the Montreal Cognitive Assessment (MoCA) is also widely used, but has a relatively narrow scoring range, appears to be too difficult for measuring the progression of dementia and is considered too long for the busy clinical practice or hospital outpatient setting [10].

The Quick Mild Cognitive Impairment screen (Qmci) is a brief screening tool designed to differentiate subjective cognitive complaints, MCI, and early dementia [11]. Compared to other CSIs, Qmci demonstrates reduced floor and ceiling effects and is quicker to administer [12]. It has been validated in several clinical settings, languages, and countries, such as Australia [13], Netherlands [14], Turkey [15], Ireland [16], China [17], Taiwan [18] and Portugal [19]. Regarding the Greek version (Qmci-Gr) it was recently adapted and translated into Greek [20].

In comparison to the MMSE and MoCA, two of the most widely used CSIs, the Qmci illustrates certain advantages in terms of accuracy, specificity, and sensitivity [21]. When the Qmci-Gr was compared with the SMMSE in Greek clinical settings, it was found that both tests were similar in detecting cognitive impairment, thereby disentangling SMC from MCI and dementia. Similar findings were reported for the MCI and dementia group [20]. Compared to the MoCA [22], a well-established CSI, Qmci appeared to be more accurate [21]. According to recent evidence [21,23], the superiority of the Qmci relies upon a highly detailed scoring system of 100 points and the Logical Memory (LM) task.

With respect to clinical practice in Greek clinical settings, particularly busy public hospital outpatient units and other respective clinical settings, there is insufficient evidence to show that suitably accurate, sensitive, and specific short cognitive screening instruments are able to differentiate MCI from normal aging and dementia. Given this important concern and considering previous findings regarding Qmci-Gr and MMSE comparisons and the frequent use of the MoCA [22] in clinical praxis, the present study investigated the differences in terms of diagnostic accuracy between the Greek version of the Qmci (Qmci-Gr) and MoCA, in discriminating older adults with SMC, MCI and dementia.

## 2. Methods

### 2.1. Participants

We recruited consecutive patients with an age range of ≥55 years from two outpatient memory clinics in Greece (University Hospital of Patras and the Nestor Psychogeriatric center, Athens, Greece) between April 2019 and September 2020. All patients underwent a detailed neurological examination, laboratory testing, comprehensive flexible neuropsychological assessment, and CT and/or or MRI scanning on a routine basis. Diagnosis was determined by an experienced consultant neurologist based on the above findings, independent of the CSIs scores. We classified patients as having dementia according to the DSM 5 criteria. The Reisberg Functional Assessment Staging scale [2] was utilized in order to establish dementia stage. Patients requiring assistance in the instrumental activities of daily living (such as handling medications and managing finances) were classified as having mild stage dementia. MCI was diagnosed on the basis of Petersen’s criteria [20], while SMC implied the presence of subjective memory complaints against a background of normal range scores upon independent neuropsychological evaluation [20]. Exclusion criteria were as follows: inability to communicate fluently in Greek, the presence of another neurological disorder and/or major psychiatric disorder, traumatic brain injury and addiction. The presence of an active depressive disorder was excluded by clinical assessment and psychometrically documented with the Geriatric Depression Scale (GDS), i.e., those scoring greater or equal to five were assessed by a trained psychologist. All participants approved their involvement by signing an informed consent form in advance. The study was approved by the Ethics Committee of the University of Patras Medical School and the Local Ethical Committee of the Psychogeriatric Association and was conducted in accordance with the principles of the Declaration of Helsinki.

### 2.2. Measures

The Q*mci* screen contains six subtests. These are (1) orientation, (2) five-word registration with immediate recall, (3) clock drawing, (4) delayed recall of words used in the registration subtest, (5) verbal fluency for categories (e.g., animals) named in one minute and (6) logical memory (immediate verbal recall of a short story). Orientation is scored out of 10 points, registration out of 5 points, clock drawing from 15 points, delayed recall from 20 points, verbal fluency out of 20 points and logical memory from a total of 30 points [12]. The Q*mci* screen therefore has a total possible score of 100 with higher scores indicating better cognition. A blank scoring template (clock face) is provided for clock drawing. The median administration time of the original English language version was approximately 4.24 min [23] and most studies have reported times of approximately five minutes [24]. The typical cut-off for CI is <62/100 rising to <67/100 for MCI (versus normal), dropping to <45/100 for dementia (versus MCI) [24]. In a Turkish sample, the optimal cutoff, for differentiating MCI from SMC was (<53), with a sensitivity of 67% and specificity of 81% [15]. In a recent study with a Persian version of the Qmci, an optimal cut-off of <53/100 was established in identifying cognitive impairment (MCI and mild AD) with a sensitivity of 88% and specificity of 80% [25].

The Greek version of the Montreal Cognitive Assessment (MoCA) is scored out of 30 points which indicates intact cognitive function. The MoCA scale assesses various cognitive domains such as short-term memory, visuospatial abilities, attention and working memory, executive functioning, language and orientation; its administration takes approximately 10 to 15 min [26].

### 2.3. Procedures

#### Translation and Adaptation

The Q*mci-Gr* screen has been previously translated and adapted into Greek via the forward–backward translation method by two health professionals who were native speakers of Greek and proficient in English, as well as an expert in Greek linguistics [see [20]]. Regarding the MoCA, the Greek-adapted version was already available in Greece [see [26]]. For the adaptation procedure, Greek words were evaluated for consistency on the following dimensions: all words were two or three-syllable concrete nouns, with no obvious semantic or phonetic associations or similarities between words in the same list. All were common words that are normally acquired in Greek speaking persons with relatively low levels of education and have relatively frequent occurrence in the Greek language. The probability of the occurrence of the word in common usage in the Greek language was ascertained by using the Institute for Language and Speech Processing Greek Corpus [27]. This Greek version was than reviewed by an expert panel of Greek health professionals (medical doctors and psychologists) and researchers and a completed version of the Q*mci*-Gr was generated. A professional, native English language-speaking translator, without knowledge of the concepts behind the screening tool, performed the back-translation. The back-translation was then reviewed by the original developers of the Q*mci* screen, who approved the final version.

### 2.4. Data Collection

In order to minimize learning effects, the Q*mci*-Gr and MoCA were administered sequentially and alternated by trained raters. One rater in each site scored the Q*mci*-Gr screen and MoCA. Raters were blind to each other and the final diagnosis. To ensure uniform test administration and scoring, qualified clinicians had previously attended training sessions. Clinicians in either site were blind to the CSI’s results. The battery of neuropsychological measures included the ‘5 Objects Test’, the Rey Auditory Verbal Learning Test, the Greek Verbal Fluency Test, the Boston Naming Test, the Frontal Assessment Battery (FAB), the Digit Span Test (forward–backwards) and the MoCA [26]. Only validated Greek versions were used. The ‘5 Objects [28] is a spatial memory (objects and locations) measure entailing an immediate (encoding) and delayed recall (retention) condition, which is also ideal for people with limited language skills given its non-verbal nature. The Rey Auditory Verbal Learning Test [see 20] is a measure of auditory–verbal memory and learning assessing encoding, consolidation, storage and retrieval. The Greek Verbal Fluency Test consists of two parts: semantic and phonemic fluency. The semantic fluency test (categories) requires the examinee to name as many different fruits or other objects as possible, each within 60 s, making use of conceptual categories other than those used in the Q*mci* screen to remedy for learning effects. The phonemic fluency test (letters) requires examinees to generate as many words as possible beginning with the Greek letters “Χ” (chi), “Σ“(sigma) and “A” (alpha), again within 60 s, excluding proper nouns and variations of the same word. The total number of words and letters produced on either test were included in the analysis [see [20]]. The Boston Naming Test assesses confrontation naming to detect anomia in dementing conditions with the number of items named correctly taken as the dependent variable [see [20]]. The FAB is a brief frontal lobe assessment tool implemented in identifying the dysexecutive phenotype in dementia [see [20]]. The test battery was administered on the same day as the screening instruments with a gap of 20–30 min between scoring. Test–retest reliability was tested at approximately two weeks.

### 2.5. Statistical Analysis

Data were analyzed using the statistical programming language R, R version 3.5.0-”Joy in Playing”. Τhe distribution of the data was assessed using the Shapiro–Wilk and Kolmogorov–Smirnov normality tests. The normality assumption was rejected for most cases; thus, non-parametric tests were utilized. The Kruskal–Wallis H test was used to compare the medians of the variables of interest between the levels of categorical variables with three or more levels. Diagnostic accuracy was evaluated by the area under the curve (AUC) receiver operating characteristic (ROC) curves, which were compared using the Delong et al., method [see [20]]. In general terms, an AUC of 0.5 suggests no discrimination (i.e., ability to diagnose patients with and without the disease or condition based on the test), 0.7 to 0.8 is considered acceptable/good, 0.8 to 0.9 is considered very good to excellent and more than 0.9 is considered outstanding.

The optimal cut-off score for each CSI was calculated using the Youden index, i.e., J = sensitivity + specificity − 1 [see [20]], such that the cut-off point optimizes the test’s ability to differentiate outcomes when an equal weighting is applied to the sensitivity and specificity.

## 3. Results

### 3.1. Demographics

A total of 145 patients were included in the analysis, from whom 69 had dementia, 32 had MCI and 44 had SMC. The median age (years) of the sample was 70 years with an interquartile range (IQR) of ±7 years. Fifty-one percent were male (74/145). The median years of education were 9 (IQR ± 6). Gender distribution and patients’ median age and time in education did not differ significantly between the three groups.

### 3.2. Test Scores

The median MoCA score for the whole sample was 24/30 (IQR ± 6) and the median Qmci-Gr screen score was 58/100 (IQR ± 24). Table 1 presents patient characteristics and their test scores.

### 3.3. Diagnostic Accuracy

The Qmci-Gr had a statistically significant lower accuracy (AUC = 0.76, 95% CI: 0.64–0.87) in distinguishing SMC from MCI patients compared with the MoCA (AUC = 0.94, 95% CI: 0.88–0.99), *p* = 0.006. The Qmci-Gr was shown to be more accurate at a borderline significance level (AUC = 0.94, 95% CI: 0.91–0.98) in distinguishing SMC from dementia, as opposed to MoCA (AUC = 0.89, 95% CI: 0.82–0.96), *p* = 0.03. Finally, the Qmci-Gr, while having a larger AUC value (AUC = 0.81, 95% CI: 0.72–0.91), displayed statistically similar accuracy in distinguishing MCI from dementia in comparison to the MoCA (AUC = 0.75, 95% CI: 0.65–0.84), (*p* = 0.08). The results of the ROC analysis are presented in Table 2.

### 3.4. Cut-Off Scores

We present optimal cutoffs for each diagnostic classification for both screening tests in Table 2. For differentiating SMC from those with MCI, applying Youden’s Index, a cutoff of <71 on the Qmci-Gr gave a sensitivity of 80% and specificity of 78%. The MoCA compared slightly favorably to this, with a sensitivity of 82% and a much higher specificity of 91% at a cutoff of <26. The optimal Qmci-Gr cut off for differentiating MCI from dementia was <55, with a sensitivity of 88% and a specificity of 79%. Comparatively, the MoCA had both a lower sensitivity (75%) and specificity (71%) at its optimal dementia cutoff of <22. One the other hand, the optimal Qmci-Gr cutoff for differentiating SMC from dementia was <53, with a sensitivity of 100% and a specificity of 75%. In this case, the MoCA also had a sensitivity of (100%), but a higher specificity (87%) at its optimal dementia cutoff of <25.

## 4. Discussion

Following the recent validation of the (Qmci-Gr) [20], the aim of the present study was to compare the Qmci-Gr to the MoCA, in respect of their diagnostic accuracy, in a Greek sample of 145 older adult patients with SMC, MCI and dementia. Here, both instruments displayed a similar accuracy in distinguishing between MCI and dementia. However, MoCA appeared to be superior in relation to its ability to discriminate between SMC and MCI, while the Qmci-Gr presented a higher accuracy in differentiating between SMC and dementia. Considering their comparable efficacy, Qmci-Gr could be proven to be practical in clinical settings where time resources are limited.

It was unexpected that MoCA exhibited a better accuracy than the Qmci-GR in differentiating SMC from MCI patients, as several researchers have mentioned better or similar results of the Qmci when compared to the MoCA [15,16]. According to [21], Qmci was superior to MoCa at detecting MCI or any other cognitive impairment when pooled results from recent studies were analyzed. In the present study, the observed difference could possibly be attributed to translation and adaptation variables inherent in the cultural and linguistic dynamics of the Qmci in the Greek population and the demographics of the present sample.

On the contrary, it was expected that both instruments would be able to discriminate MCI from dementia groups in a similar way; however, the Qmci demonstrated a better AUC curve, even though it was marginally statistically significant. Similar findings were noted from the Australian comparison of these CSIs [13]. Specifically, Qmci was more accurate in distinguishing MCI from dementia or controls, but no statistical significance was found. The Qmci was also more balanced regarding sensitivity and specificity in discriminating normal cognition from MCI compared to the MoCA in their same sample [13]. In an Irish sample, the Qmci separated normal cognition from MCI more accurately than the MoCA [16].

With regard to the diagnostic accuracy between SMC and dementia, although both instruments exhibited excellent sensitivity and specificity, the Qmci was proven to be more accurate than the MoCA. This finding verifies the outcomes from limited previous studies. In the comparison of the two diagnostic instruments in Irish clinical settings, the Qmci demonstrated a better, though non statistically significant, accuracy [16]. Likewise, in a Turkish sample [15], the Qmci displayed a higher sensitivity in differentiating SMC from dementia, but only regarding younger patients (≤75 years) with a higher educational level (≥8 years). However, due to the confined number of studies including SMC subjects, Qmci’s superiority regarding these groups should be further investigated.

The observed Qmci-Gr cutoffs resulting from Youden’s index differ from those suggested in the Greek validation study [20]. Specifically, for the present sample, the optimal cutoff to distinguish SMC from MCI was <71/100 and from dementia <53/100, while the validation study showed that the optimal cutoff for discriminating SMC from a group of MCI and early dementia patients combined was <51 [20]. According to [24], a score of <62 reflected the optimal sensitivity and specificity for detecting cognitive impairment (MCI or dementia). The optimal score for the MoCA, indicating objective cognitive decline and thus differentiating SMC from MCI, was a cut off score of 26 (Youden index = 0.72). For diagnosing dementia, a cutoff of 25 points was established (Youden index = 0.87). These scores are relevant to the cut-off score of 26 suggested by [22] for cognitive impairment. Recent studies [11,13,29], conducted in different countries, indicated that the optimum cutoffs for detecting cognitive impairment in diverse populations were set lower than that suggested originally by [22]. Lower cut-off scores were also presented in recent meta-analyses [30,31]. However, the MoCA cutoffs, as defined in this study, are consistent with those suggested by [26] regarding the normative data for MoCA in Greek older adults, signifying that the optimal cut-off scores may depend on the distinct demographic characteristics of the sample.

The study, despite its strengths, presents several limitations. Our sample was relatively small and restricted to patients attending only two Greek outpatient clinics. Therefore, our clinical sample may not be representative of all Greek older adults eligible for cognitive assessment. The small sample may have attenuated the chance to find significant results regarding the diagnostic superiority of a CSI. Additionally, more SMC and MCI patients are needed, as the majority were dementia patients, limiting the generalizability of the results. Another limitation is that the SMC subgroup had slightly more years of education (although non significantly different) compared to MCI and dementia patients. It has been reported that years of education affect performance and the cut-off scores for both Qmci [24] and MoCA [30,32,33], although a higher cognitive reserve assumed for those with higher education probably justifies their normal cognition. This could explain, to a certain extent, the results regarding the SMC group.

## 5. Conclusions

In conclusion, the present results suggest that the Qmci has comparable diagnostic accuracy to the MoCA in these settings, especially with regard to MCI and dementia groups. Based on this sample, a score of 71/100 or less suggested cognitive impairment, while the equivalent cutoff for MoCA was 26/30, as suggested by [22]. In order to assign a specific cut-off score, below which further cognitive evaluation will be required, it is crucial to examine all the psychometric properties of the instrument. A larger, more diverse sample could help eliminate the aforementioned limitations. Given its brief nature and similar accuracy with widely used CSIs in Greece, it is possible that the Qmci-Gr will earn a place in everyday clinical practice.

## Figures and Tables

**Table 1:** Characteristics of the patients—total and by diagnostic classification.

Characteristics	Total (*n* = 145)	SMC (*n* = 44)	MCI (*n* = 32)	Dementia (*n* = 69)	*p*-Value
Age					
Median years (±IQR)	70 (±7)	69 (±6)	71 (±4)	71 (±12)	0.088
Gender					
Proportion female (%)	49%	56.8%	46.9%	44.9%	0.451
Time in education					
Median years (±IQR)	9 (±6)	10 (±6)	7 (±4)	9 (±6)	0.053
Qmci-Gr screen					
Median (±IQR)	58 (±24)	74 (±12)	61 (±7)	49 (±7)	<0.005 *
MoCA					
Median (±IQR)	24 (±6)	26 (±2)	24 (±4)	20 (±8)	<0.005 *

IQR = interquartile range; MCI = Mild Cognitive Impairment; Qmci-Gr screen = Quick Mild Cognitive Impairment screen—Greek version; SMC = Subjective Memory Complaints; MoCA = Montreal Cognitive Assessment. * Statistically significant, *p* < 0.05.

**Table 2:** Sensitivity and specificity, Youden’s Index (J) and area under the curve (AUC) scores with cut-off scores for the Quick Mild Cognitive Impairment screen—Greek version (Qmci-Gr) compared with the Montreal Cognitive Assessment. (MoCA), without adjustment for age, gender, and education.

Diagnostic Classification	Cutoff	Youden’s Index (J)	Sensitivity	Specificity	AUC (95% CI)
Qmci-Gr					
SMC from MCI	<71	0.58	80%	78%	0.76 (0.64–0.87)
MCI from dementia	<55	0.67	88%	79%	0.81 (0.72–0.91)
SMC from dementia	<53	0.75	100%	75%	0.94 (0.91–0.98)
MoCA					
SMC from MCI	<26	0.72	82%	91%	0.94 (0.88–0.99)
MCI from dementia	<22	0.46	75%	71%	0.75 (0.65–0.84)
SMC from dementia	<25	0.87	100%	87%	0.89 (0.82–0.96)

CI = confidence interval; MCI = Mild Cognitive Impairment; SMC = Subjective Memory Complaints but normal cognitive testing.

## Data Availability

Data can be obtained by the corresponding author upon request.
